# Mitigating bus bunching with real-time crowding information

**DOI:** 10.1007/s11116-022-10270-3

**Published:** 2022-03-04

**Authors:** Arkadiusz Drabicki, Rafał Kucharski, Oded Cats

**Affiliations:** 1 Department of Transportation Systems, Cracow University of Technology, Kraków, Poland; 2 Group of Machine Learning Research, Jagiellonian University, Kraków, Poland; 3 Department of Transport and Planning, Delft University of Technology, Delft, The Netherlands

**Keywords:** Public transport, Overcrowding, Bus bunching, Real-time crowding information, RTCI, Willingness to wait

## Abstract

A common problem in public transport systems is bus bunching, characterized by a negative feedback loop between service headways, number of boarding passengers and dwell times. In this study, we examine whether providing real-time crowding information (RTCI) at the stop regarding the two next vehicle departures can stimulate passengers to wait for a less-crowded departure, and thus alleviate the bunching effect. To this end, we leverage on results from own stated-preference survey and develop a boarding choice model. The model accounts for the presence of RTCI and is implemented within dynamic public transport simulation framework. Application to the case-study model of a major bus corridor in Warsaw (Poland) reveals that RTCI can induce a significant probability (30–70%) of intentionally skipping an overcrowded bus and waiting for a later departure instead. This behaviour, in turn, results in significantly lower vehicle headway and load variations, without deteriorations in total waiting utility. Overall, journey experience improves by 6%, and crucially—the prevalence of denial-of-boarding and excessive on-board overcrowding is substantially reduced, by ca. 40%. Results of our study indicate that the willingness to wait induced by RTCI can be a potential demand management strategy in counteracting bunching, with benefits already attainable at limited RTCI response rates.

## Introduction

Reliability of public transport (PT) operations is an important aspect for the perceived PT quality of service, which is profoundly influenced by variations in travel times and on-board travel conditions. In this context, variability of passenger flows is one of the key determinants of PT service reliability (Sorratini et al. 2008), especially in urban, congested transport networks. Excessive, unstable demand flows can ignite supply performance perturbations that can quickly spiral from an isolated event into a network-wide disruption (Cats et al. 2011b), and exacerbate service disruptions even without exogenous disturbances, posing major risks in low and high demand conditions (Fonzone et al. 2015).

A clear illustration of this inherent PT system instability is the so-called bus bunching effect (Newell and Potts 1964; Cats et al. 2011b). Bunching is the consequence of a negative feedback loop between service headways, number of arriving passengers and dwell times. The bus bunching pattern is likely to become self-amplified along the service line, as the first bus becomes increasingly overcrowded and delayed at downstream stops, while the second bus is underutilized and ‘catches up’ with the predecessor, leading to their ‘platooning’ in extreme cases (Yu et al. 2016). This phenomenon results in unreliable services, with a series of negative implications for passengers and operators—longer journey times, service delays, higher travel discomfort, risk of overcrowding and denied boarding, uneven headway and load distribution and an inefficient capacity utilization (Cats et al. 2016). A major contributor to the bunching problem is passengers’ inherent preference towards boarding the first available departure, which exacerbates further the crowding—bunching feedback effect. Therefore, strategies aimed at stimulating *co-operative* boarding behaviour amongst passengers—presumably through the provision of real-time crowding information—shall be explored as a potential measure to counteract the bus bunching (Delgado et al. 2012; Wu et al. 2017).

### Literature review

To counteract the undesirable bunching effect, various tactical operational strategies have been conceived. On the supply side, these involve holding control strategies [commonly evaluated as an optimized weighted function of passenger travel (dis)utility and/or the closed-form function of bus arrival times, e.g.: Adamski and Turnau 1998; Cats et al. 2011b; Bartholdi and Eisenstein 2012; Delgado et al. 2012; Berrebi et al. 2015; Berrebi et al. 2018; Laskaris et al. 2019; Gkiotsalitis and Cats 2019; Gkiotsalitis and van Berkum 2020; Wang and Sun 2020]; allowing for buses to overtake each other (Sun and Schmöcker 2018; Schmöcker et al. 2016; Wu et al. 2017); stop-skipping (expressing) (Fu et al. 2003; Sun and Hickman 2005; Larrain and Muñoz 2020); short-turning (Zhang et al. 2017; Leffler et al. 2017, Gkiotsalitis et al. 2019); rescheduling (Gkiotsalitis 2019); speed adjustment (Daganzo and Pilachowski 2011); and robust slack-time planning (Zhao et al. 2016). Another, relatively less-explored stream of research is devoted to impacts of demand-side phenomena in managing the bus bunching problem, albeit with limited empirical underpinning (Wu et al. 2017; Sun and Schmöcker 2018; Wu et al. 2019).

A series of works have dealt with the notion of applying boarding limits to counteract the bunching effect. Imposing boarding limits or a no-boarding policy implies that when specific (in)stability criteria are met, the PT operator constrains the number of passengers allowed to board a PT vehicle, forcing the remaining ones to wait at the stop for a later departure. In the context of reversing the bus bunching mechanism, while holding strategies aim at ‘slowing down’ the second bus that moves ahead of schedule, the objective of no-boarding strategies is to ‘speed up’ the first bus that is otherwise increasingly delayed (Saw et al. 2019). Delgado et al. (2012) estimate that boarding limits can foster the benefits of holding strategies when applied simultaneously, particularly in the case of high-frequency and high-demand services, requiring fewer and shorter holding times to restore service regularity and travel comfort. Boarding limits implemented as a sole (isolated) measure were also found to be effective in reducing headway variability and improving travel times (Zhao et al. 2016; Enayatollahi et al. 2019). Saw et al. (2019) conclude that no-boarding policies perform favorably in mitigating the bus bunching when compared against holding strategies in busy periods, but contrarily—backfire during low-demand periods (by imposing excessive waiting times) when holding is a more advantageous solution.

Aside from the notion of fixed boarding limits, a number of papers analyse whether shifts in passengers’ boarding behaviour can also help mitigate bus bunching. Wu et al. (2017) introduce a dynamic queue swapping behaviour model, where boarding probability is proportional to the available (residual) capacity of next bus arrivals—i.e. passengers at a stop form *self-equilibrating* boarding queues for the first and second incoming departures. They found that in conjunction with enabling overtaking between buses, this brings substantial performance and travel experience benefits, regardless of network demand levels and without employing any holding controls. Sun and Schmöcker (2018) demonstrate that in the event of bus bunching, a hypothetically high preference towards waiting deliberately for a second bus is in general advantageous for restoring the service regularity and improving travel experience, even without additional control measures.

Studies acknowledge that favourable shifts in passengers’ boarding behaviour may be encouraged by providing information on the in-vehicle crowding levels (Delgado et al. 2012; Palma et al. 2015; Schmöcker et al. 2016; Wu et al. 2017). This is an increasingly feasible solution as data on past and current passenger flows in PT networks is gathered via a variety of sources—including Automated Passenger Counts (APC) and Automated Fare Collection (AFC) systems, video recording data, remote sensing, mobile and wireless networks, and most importantly—smart card ticketing systems. These data can be processed and conveyed to passengers in the form of real-time crowding information (RTCI) (Jenelius 2020). Current state-of-the-practice of RTCI systems involves usually pilot and limited-scale projects, ranging from localised crowding information on individual train carriages’ loads (e.g. London (Schmitt 2017), Sydney (Susan 2017), Tokyo (EJRC 2019), information on bus occupancy loads (e.g. Seoul (SMC 2017) and train crowding [e.g. Stockholm Metro (Zhang et al. 2017), Dutch Railways (NS 2021)], to network-wide information on expected crowding levels of PT routes, available via travel apps [e.g. Google Maps (Google LLC 2021), Moovit (Moovit Inc 2021), JakDojade.pl (City-Nav LLC 2021)]. Recently, RTCI provision has been gaining momentum as operators aim to tackle the ramifications of the (on-going) covid-19 pandemic crisis for perceived travel safety. This has been witnessed in several systems in the US, e.g. in Boston (MBTA 2021), San Jose (VTA 2021) and Washington D. C. (WMATA 2020), where crowding information is disclosed in real-time to reassure travellers about on-board crowding conditions and the possibility to maintain social distancing.

Access to crowding information concerning the next PT trips (departures) at a stop can induce a specific decision pattern among passengers, namely the willingness to wait (WTW) to reduce (or avoid) on-board overcrowding experience—i.e., by skipping the first (overcrowded) trip and waiting at the stop to board a second, less-crowded trip later. This phenomenon has been estimated in a number of stated preference (SP) studies by means of survey questions (Kim et al. 2009; Kroes et al. 2014) or choice experiments (Preston et al. 2017; Kattan and Bai 2018; Drabicki et al. 2022). These studies found that the extent to which passengers are willing to wait is mainly driven by high overcrowding levels in the first departure, where the WTW tends to rise non-linearly above a perceived discomfort threshold, and on the waiting time for the second departure from the same PT stop. Average acceptable waiting times were found to oscillate between: ca. 3–12 [mins]—for urban PT (bus and tram) systems (Drabicki et al. 2022), and ca. 8–25 [mins]—for regional rail trips (Preston et al. 2017). In the case of urban PT trips, a 5-min wait is deemed acceptable for 40% of respondents—if the first bus/tram is moderately crowded (i.e. has ‘comfortable’ standing space available), and even for up to 80% of them—if it is severely overcrowded (i.e. possible denied-boarding risk). Survey findings from a Paris metro system report analogous figures, ranging from 12% (first train—minor crowding) to 75% (first train—severe overcrowding) (Kroes et al. 2014).

The potential network-wide impact of RTCI upon passengers’ travel behaviour has been assessed by several studies using PT simulation models. These studies focus on specific RTCI consequences and spatial (i.e., route choice) or temporal (i.e., departure choice) impact dimensions. Bouman et al. (2016) develop a game-theory based model and analyse crowding information provision in context of the so-called El Farol Bar problem. Toy network simulations underline its potential to enhance capacity utilization, but also the risk of lower payoffs with rising network coverage and responsiveness to crowding information. Nuzzolo et al. (2016) proposed a mesoscopic PT assignment model that simulates the impact of predictive RTCI on long-term (day-to-day) travel decisions. Predictive RTCI and network assignment are achieved as an iterative outcome of in-vehicle passenger loads merging towards choice probabilities. Case study results show that long-term adaptation to such crowding prediction induces substantial departure time shifts in everyday journeys (e.g., AM peak widening), and a certain decrease in waiting times and denied-boarding risk. Noursalehi et al. (2019) introduce a dynamic, mesoscopic PT assignment model for analysing the impacts of localized (station-level) predictive RTCI on instantaneous departure choices of next train departures. Crowding prediction refers here to the probability of being able to board an arriving PT vehicle (i.e., guaranteed/likely/unlikely). Likewise, it is estimated in a rolling horizon approach in 15- to 30-min intervals as an iterative outcome of convergence between passengers’ anticipations, disseminated RTCI content and actual travel condition. Application findings reveal that passengers’ acceptance of boarding deference (i.e. WTW rate induced by predictive RTCI) eventually leads to much lower overcrowding experience and improved on-board comfort. Drabicki et al. (2021b) develop a modelling framework of instantaneous RTCI effects upon route (path) choices. The model, embedded within BusMezzo, a dynamic transit assignment simulation model, assumes that RTCI is generated based on latest reported crowding levels of downstream line segments, and then utilized by passengers to evaluate route choice probabilities. Model demonstration reveals that such crowding information can improve travel experience—in particular, by reducing the incidence of worst overcrowding experience—but it is also marked by substantial inaccuracy risks, especially on the crowding underestimation side. Finally, a recent study of Wang et al. (2021) presents the toy-network simulation of impacts of passenger boarding shifts in response to RTCI on bus crowding loads. Demonstration of their algorithm suggest improvements of ca. 20% in terms of bus headways and bus run times and its potential to coordinate with holding control strategies. The policy performance is seemingly robust even with low WTW rates among the passengers.

In summary, although state-of-the-art studies have explored the prospects of demand-side interventions in managing the bus bunching, they predominantly involved *centralized*, *top-down* assumptions on operators’ boarding policies (fixed boarding limits) or passengers’ boarding choices (fixed splitting rates or self-equilibrating queuing behaviour). In contrast, the potential ramifications of *decentralized*, *individual* boarding decision patterns that might be stimulated by provisioning RTCI are not yet fully understood. Arguably, there is still a lack of comprehensive and evidence-based knowledge on collective dynamics, which might emerge from more informed individual decision-making in the context of bus bunching.

### Objective and contribution

The objective of this study is to assess the effects of real-time crowding information (RTCI) on travel experience and service reliability in the context of bus bunching problem. To this end, we model the passengers’ instantaneous boarding choices with access to RTCI on crowding levels of next two PT departures from the current stop. The choice model is developed based on own stated-preference empirical findings of the willingness to wait (WTW) behaviour with RTCI (Drabicki et al. 2022). It is then implemented within a dynamic, agent-based PT assignment model, reproducing its wider impacts upon PT system performance and journey experience.

The potential of RTCI in mitigating the bunching is demonstrated through an application to a case study of a busy bus corridor in Warsaw (Poland). As shown by our results, the WTW with RTCI can greatly mitigate the evolution of bus bunching, prompting up to 30–70% of passengers to wait voluntarily for a less-crowded departure. Overall, this results in major improvements in terms of service performance, journey experience and eliminated risk of ‘full bunching’ phenomenon. The supply side witnesses lower headway variations and more uniform vehicle loads’ distribution. Benefits for passengers are evident across the majority of bus stops, especially at central and downstream parts of the service, where further evolution of bus bunching is effectively eliminated. Interestingly, WTW does not necessarily result in longer total waiting time. Consequently, RTCI provision leads to global decline in both nominal journey times (2%) and generalized travel disutility (5%), and substantially reduced risks of in-vehicle overcrowding and denial-of-boarding (40%). Travel experience benefits are attainable already with limited responsiveness to RTCI, while higher RTCI utilization among passengers can also foster the service regularity.

This work aims to contribute to the (insofar limited) state-of-the-art stream of research on the prospective role of RTCI in mitigating bus bunching. Initial literature findings (Wang et al. 2021) have illustrated the value of RTCI in regulating bus services for numerical, toy-network simulations. We extend these considerations in our study and develop the PT operations and assignment model, underpinned by empirical WTW sensitivity analysis. Case study application reveals plausible RTCI effects on a real-world bus corridor model, whose overall benefits are captured via multiple metrics. We also investigate the impact of various level of responsiveness to the information provisioned. We conclude this study with discussion on our findings and the prospects of RTCI in real-time service and demand management strategies.

The remainder of this paper is organized as follows. Section “Method” presents the methodology of the proposed WTW choice model with RTCI and its embedment within a dynamic PT simulation model. Section “Application and Results” follows with application results of the proposed simulation model to a bus corridor case study network. We conclude this study with Sect. “Discussion”, containing a summary of findings and practical implications.

## Method

The following section presents the methodological framework of our research investigation of RTCI impacts on bus bunching effect. We begin with description of modelling inputs, requirements and the dynamic PT assignment model used in this study. Then, we present the passenger departure choice algorithm, simulating the impact of instantaneous RTCI on instantaneous boarding decisions at stops and further ramifications for PT system performance.

### Modelling requirements

Capturing the interactions between crowding levels, real-time information and bus bunching requires a simulation framework that models the PT system in an explicit, disaggregate way—with PT demand represented by individual agents (travellers) and PT supply model described by individual vehicles (trips). The PT model shall capture how interactions between the PT demand (i.e. passenger overcrowding) and PT supply (i.e. bus bunching) may evolve dynamically and amplify each other. This pertains especially to the real-time feedback between passenger flows, dwell times and service headways. In addition, several effects of passenger overcrowding need to be properly reproduced: the influence of on-board passenger loads upon rising travel discomfort; the effects of strict capacity constraints and possible denial-of-boarding effect; and resultant consequences in form of uneven load distribution and service regularity.

Another modelling requirement relates to the working principles of RTCI systems in PT networks. Firstly, how the crowding information is recorded from instantaneous PT data on passenger loads in the PT vehicles. Secondly, the mapping of ‘raw’ crowding data into the provision of a user-friendly RTCI. Thirdly, how the generated RTCI information is instantaneously disseminated at PT stops and stations across the network.

Finally, on the demand side, the modelling framework shall represent the RTCI impacts on passengers’ dynamic decision-making process. This includes the acquisition of RTCI at the stop(s); ubiquitous (system-wide) reaction to RTCI content; the incorporation of RTCI input into individuals’ boarding choice; and the resultant travel decision, i.e. boarding now vs. willingness to wait for next departure.

### Simulation model environment—BusMezzo

For the purposes of our analysis, we will use the *BusMezzo* mesoscopic, agent-based PT simulation model (Cats 2011). The *BusMezzo* model assumes an explicit, disaggregate representation of PT system, which enables in-depth analysis of the PT overcrowding (Cats et al. 2016) and PT capacity reductions’ (Cats and Jenelius 2018) effects on passengers’ travel experience and network performance. It has been also applied for analysing the impacts of real-time information (RTI) concerning subsequent vehicle arrivals on travel times (Cats et al. 2011a; Cats and Jenelius 2014) and real-time crowding information (RTCI) on instantaneous route choices (Drabicki et al. 2021b). Furthermore, *BusMezzo* is a dynamic assignment model, where PT network performance is an outcome of sequential and mutual interactions between supply side (individual vehicle movements) and demand side components (passengers’ travel actions). Consequently, it is also applicable for investigating the bus bunching phenomena (Moreira-Matias et al. 2016) and holding and control strategies (Cats et al. 2011b; Laskaris et al. 2019; Gkiotsalitis and Cats 2019).

The PT network model in *BusMezzo* is represented in form of directed, connected graph *G*(*S,E*), comprising a set of nodes (stops) *S*, and a set of links *E*. The latter consists of line (trip) segments and walking connections (access, egress and transfer links). The line segment *e* of a PT line *L* connects its tail stop ${e}^{-}\in S$ with its head stop ${e}^{+}\in S$.The PT line *L* is an ordered sequence of vehicle trips (runs) $r,\,r+1,...\in L$ serving a sequence of stops *L* = (*s*_*0*_, *s*_*1*_, *s*_*2*_*,* …, *s*_*n*_), or equivalently, operating along a sequence of line segments *L* = (*e*_*0*_, *e*_*1*_, *e*_*2*_*,* …, *e*_*n-1*_).

The *BusMezzo* model enforces strict capacity constraints (Cats et al. 2016; Gavriilidou and Cats 2019). Each vehicle *r* is characterized by its respective seat capacity *ƞ*_*r*_ and total (crush) capacity *κ*_*r*_. This allows to account for three principal categories of (over)crowding effects. First, the impact of increasing volume-capacity ratio that implies higher in-vehicle travel discomfort (disutility), especially for standing passengers. Second, passenger volumes *K*_*r,e*_^*c*^ that exceed the vehicle crush capacity limit *κ*_*r*_ are eventually left behind at stop. This triggers a further chain of events and increases the boarding volume of the next incoming departure *r* + 1*.* Third, these changes in passenger flows directly affect the PT service regularity due to flow-dependent dwell times. Vehicle dwell times *t*_*r,s*_ at stop *s* are a function of the total number of boarding and alighting passengers, *K*_*r,s*_^*b*^ and *K*_*r,s*_^*a*^, respectively, plus the number of passengers on-board *K*_*r,s*_^*c*^ of the vehicle run *r* (TCRP 2013). In this study, we consider the case where boarding and alighting is allowed via multiple bus doors, and more precisely—two set of doors per bus and uniform split of boarding and alighting flows (i.e. 50% of passengers per door). Boarding times are affected by the on-board crowding conditions. Given such settings, the dwell time *t*_*r,s*_ is given by the following formula (Eq. 1):

1 $${t}_{r,s}=\frac{1}{2}\cdot \left[{K}_{r,s}^{b}\cdot ({\tau }_{r}^{b}+{\tau }_{r}^{b,crowd})+{K}_{r,s}^{a}\cdot {\tau }_{r}^{a}\right]$$

In this study, we assume dwell-time increments of *τ*_*r*_^*b*^ = 2.0 [s/pass.] for boarding flows and *τ*_*r*_^*a*^ = 1.5 [s/pass.] for alighting flows, respectively. Moreover, an extra boarding time penalty of *τ*_*r*_^*b,crowd*^ = 2.0 [s/pass.] applies if on-board passenger volume exceeds the seated capacity, i.e. *K*_*r,s*_^*c*^ > *ƞ*_*r*_ (and equal zero otherwise). This is to reflect the impact of bus overcrowding on rising dwell times (and more often on boarding times), which has been noted in the literature (Tirachini et al. 2013; TCRP 2013; Gentile and Noekel 2016).

Total operating time of a trip *r* is the sum of running times *t*_*r,e*_ (which are assumed constant) along line segments $e\in L$ and dwell times *t*_*r,s*_ (which are flow-dependent) at line stops $s\in L$. Any fluctuations in dwell times between consecutive trips *r*, *r* + *1* influence their actual headway *h*_*r,e*_ further downstream. This allows for reproducing the classical bus bunching pattern, i.e. the feedback loop between dwell times *t*_*r,s*_, trip headways *h*_*r,e*_ and boarding volumes *K*_*r,s*_^*b*^ that is likely to become self-amplified at next downstream stops.

The PT demand model is represented by the OD (origin–destination) passenger matrix, consisting of *K*_*o,d*_—passenger rates (per hour or other time period) generated at constant inflow rates at the origin stop *o* and travelling to the destination stop *d.* While progressing through the PT network, each passenger *k* makes a sequence of travel decisions, choosing one of the possible travel actions ${a}_{k,s}\in A$ at each decision point *s*. The choice model is based on the utility maximization principle, and the choice probability *p*_*k,s*_ is given by the probabilistic MNL formula (Eq. 2), which evaluates the action utility *u*_*a,k,s*_ against the complementary utility of remaining choice (action) set *A’:*

2 $${p}_{{a}_{k,s}}=\frac{\mathit{exp}\left({u}_{{a}_{k,s}}\right)}{{\sum }_{A{^{\prime}}\epsilon A}\mathit{exp}\left({u}_{A{^{\prime}}}\right)}$$

Taking an action *a* is associated with travelling along a path *i* belonging to path set *I*_*a*_, and action utility *u*_*a,k,s*_ is calculated as the logsum of path utilities *u*_*i,k,s*_ (Eq. 3):

3 $${u}_{a,k,s}=\mathit{ln}{\sum }_{i\in {I}_{a}}\mathit{exp}\left({u}_{i,k,s}\right)$$

Each path is a sequence of stops (or equivalently, line segments) between the current decision point *s* and the destination stop *d,* i.e. *i* = (*s*, *s*_*1*_, *s*_*2*_*,* …, *d*)*.* The path set *I*_*a*_ contains all the possible and relevant travel alternatives between *s* and *d*, aside from paths which do not fulfill logical constraints (e.g. getting off and on the same bus again, for details see Cats et al. (2011a)). The path utility *u*_*i.k,s*_ is the sum of systematic path utility *v*_*i,k,s*_, and a random error term *ε*_*k*_ (Eq. 4). The latter is described by normal distribution (with mean value equal to zero) and aims to reflect utility perception (taste) differences among passengers $k\in K$:

4 $${u}_{i,k,s}={v}_{i,k,s}+{\varepsilon }_{k}={\sum }_{e\in i}{\beta }_{e,k}^{x}\cdot {t}_{e}^{x}+{\varepsilon }_{k}$$

The systematic part of utility, *v*_*i,k,s*_ is the sum of expected travel utility of path components *x*, i.e. travel time attributes *t*_*e*_^*x*^ and other trip attributes *n*_*e*_^*x*^, multiplied by their relative perceived (dis)utility coefficients *β*_*e*_^*x*^. The utility function includes the expected (perceived) travel (dis)utility related to the in-vehicle travel times *t*_*e*_^*ivt*^, wait times *t*_*e*_^*wt*^ and walk times *t*_*e*_^*wkt*^ of all trip segments *e ϵ i*, plus the (dis)utility due to the number of transfers *n*_*i*_^*tr*^ required along the path *i* (Eq. 5):

5 $${v}_{i,k,s}={\sum }_{e\in i}{\beta }_{e}^{ivt}\cdot {t}_{e}^{ivt}+{\sum }_{e\in i}{\beta }_{e}^{wt}\cdot {t}_{e}^{wt}+{\sum }_{e\in i}{\beta }_{e}^{wkt}\cdot {t}_{e}^{wkt}+{\beta }^{tr}\cdot {n}_{i}^{tr}$$

The baseline values for perceived disutility coefficients of wait time *β*_*e*_^*wt*^ and walk time *β*_*e*_^*wkt*^ are assumed to be equal twice the perceived disutility coefficient rate of uncrowded in-vehicle travel time, i.e. *β*_*e*_^*ivt*^ = − 1.0 and *β*_*e*_^*wt*^ = *β*_*e*_^*wkt*^ = − 2.0, as suggested in the literature (Cats et al. 2016; Gentile and Noekel 2016). Perceived transfer disutility coefficient β^tr^ is assumed to be equivalent to extra 5 min of the uncrowded in-vehicle travel time, i.e. every transfer imposes additional disutility equal to the *β*_*e*_^*ivt*^ = − 1.0 multiplied by 5 min. Since all perceived utility coefficients are negative, the resultant path utility is a negative value.

Crucially, with access to RTCI, the *β*_*e*_^*x*^ coefficients become a function of the information provisioned, as passengers evaluate trade-off(s) between waiting time vs. total path (dis)utility based on instantaneous crowding information available at a given stop. In the following subsection, we describe the modelling extension of the *BusMezzo* framework that allows for capturing the effects of RTCI upon passengers’ instantaneous boarding choices.

### Model extension—willingness to wait with RTCI

**Fig. 1:**
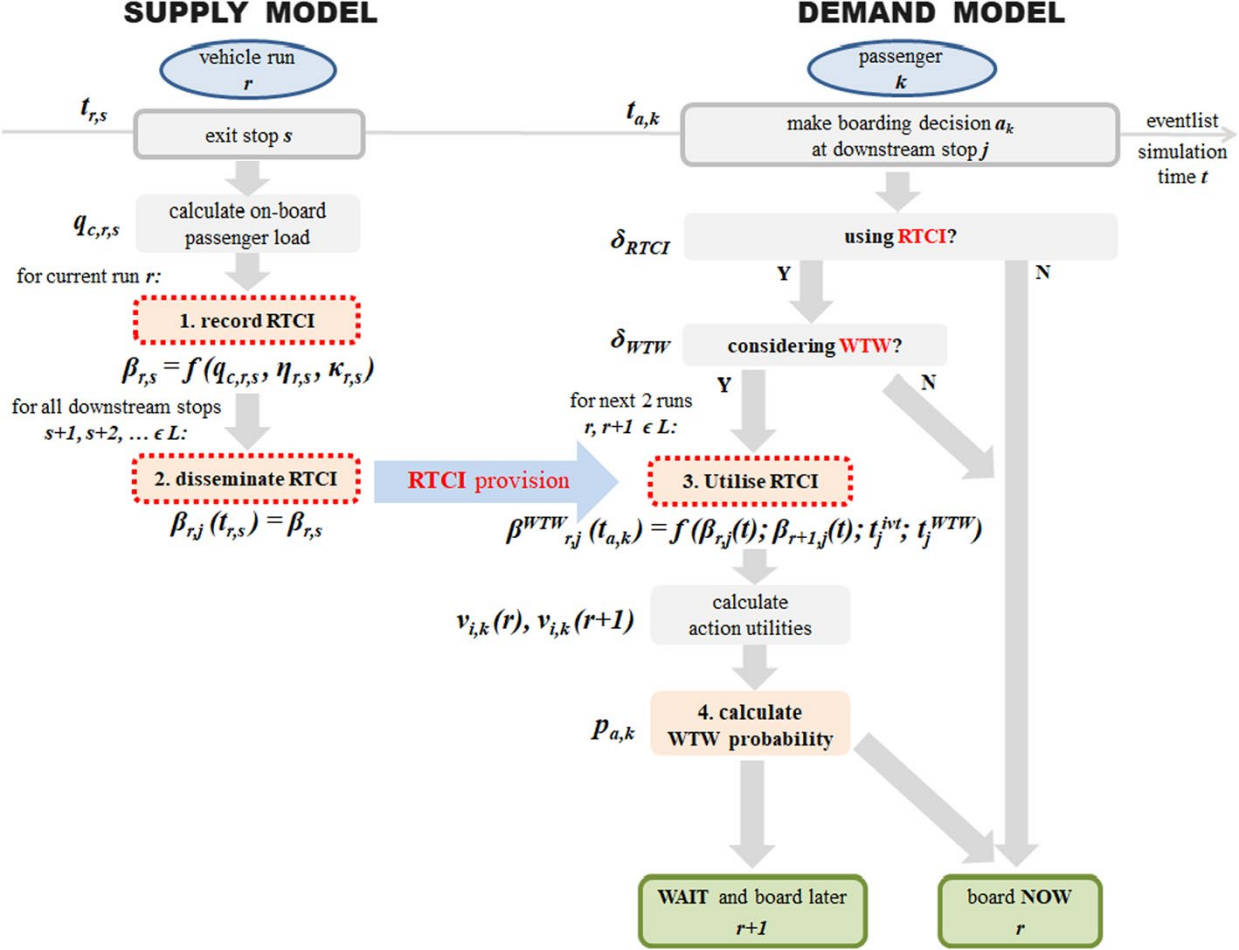
Framework of the proposed instantaneous departure (WTW) choice model with RTCI

We build on the modelling framework presented in Drabicki et al. (2021b) and proof-of-concept analysis in Drabicki et al. (2021a), extending them to cover the broad spectrum of corridor-specific dynamics of boarding decisions. We model the RTCI effects on boarding choices as part of a dynamic PT assignment model with a three-step framework (Fig. 1):

1. Recording (observing) the crowding information of PT vehicles as they depart from upstream stop(s).
2. Generating the real-time crowding information (RTCI) from instantaneous crowding data and its dissemination at downstream stop(s).
3. Incorporating RTCI into passengers’ boarding decisions.

In line with our empirical survey design (Drabicki et al. 2022), we assume that RTCI system conveys information on the two nearest vehicle departures from a given stop, containing their (expected) arrival times and on-board crowding levels.

The key variable affecting the boarding choice model with RTCI is *β*_*r,s*_^*RTCI*^—the run-specific in-vehicle passenger crowding rate, valid at a given stop *s.* This value denotes the information on the in-vehicle (over)crowding conditions, plotted on a 1-to-4 scale (Tables 1 and 2). We use this parameter to describe the working principles of the RTCI system across the following stages:

1. *Recording the RTCI* At each stop-exit instance *t*_*r,s*_, when a vehicle run *r* departs from an upstream stop *s*, its crowding level *β*_*r,s*_ is recorded. It is computed as a value on an ordinal scale (1–4) according to the mapping procedure described in Tables 1 and 2 below, as a function of: current on-board passenger load *K*_*r,s*_^*c*^, vehicle seat capacity *ƞ*_*r*_ and total (crush) capacity *κ*_*r*_ (Eq. 6):
  6 $${\beta }_{r,s}=f\left({K}_{r,s}^{c},{\eta }_{r},{\kappa }_{r}\right)$$
2. *Generating the RTCI* At each stop exit instance *t*_*r,s*_, the newly recorded RTCI is used to update the current RTCI *β*_*r,j*_(*t*) of vehicle run *r* that is instantaneously disseminated to passengers at all downstream stops *j* = *s* + *1*, *s* + *2*, *…* of line *L* (Eq. 7):
  7 $${\beta }_{r,j}\left({t}_{r,s}\right)={\beta }_{r,s}\text{ ; }\forall j=s+1,s+2,...\in L$$
3. *Utilising the RTCI* At each boarding decision instance *t*_*a,k*_, when the vehicle run *r* arrives at the downstream stop *j*, we assume that passenger *k* acquires the currently generated RTCI on two subsequent vehicle departures *β*_*r,j*_(*t*) and *β*_*r*+*1,j*_(*t*). We adopt the empirical findings from own stated-preference survey to specify the so-called willingness to wait (WTW) threshold, *t*_*r,j*_^*WTW*^. For each combination of crowding levels of these two vehicle departures, we determine the average acceptable waiting time *t*_*r*+*1,j*_^*wt*^ for next departure *r* + 1*,* in exchange for (expected) reduction in on-board (over)crowding levels between *β*_*r,j*_(*t*) and *β*_*r*+*1,j*_(*t*), as displayed by the RTCI system (Eq. 8). The WTW thresholds from the stated-preference study are presented in Table 2 and used in Eq. 8 as follows:
  8 $${t}_{r,j}^{WTW}=f\left({\beta }_{r,j}\left({t}_{a,k,j}\right),{\beta }_{r+1,j}\left({t}_{a,k,j}\right)\right)$$
  Based on these, each passenger evaluates her/his WTW utility coefficient ***β***_***r,j***_^***WTW***^**(*t*)**. Analogous to Preston et al. (2017), we obtain this rate as a function of the WTW threshold *t*_*r,j*_^*WTW*^ and the remaining (in-vehicle) travel time *t*_*e*_^*ivt*^ between the current stop *j* and the downstream alighting stop. Analogous to other path utility coefficients (Eq. 5), the WTW coefficient is evaluated in negative values (Eq. 9):
  9 $${\beta }_{r,j}^{WTW}\left({t}_{a,k,j}\right)=-1\cdot \left(1+\frac{{t}_{r,j}^{WTW}}{{\sum }_{e\in i}{t}_{e}^{ivt}}\right)$$
  The WTW coefficient acts as an additional travel disutility factor due to (expected) overcrowding in deciding whether to board the first run *r* or skip it and wait for the second run *r* + 1. When vehicle run *r* approaches the downstream stop *j*, this instantaneously triggers the WTW-based boarding decision process among waiting passengers. Each passenger *k*, if he/she has access to RTCI, chooses between the boarding utility *v*_*i.k,j*_(*r*) vs. staying utility *v*_*i.k,j*_(*r* + 1). Here, we set the staying utility, associated with taking the later departure *r* + 1*,* as reference value equal to the (negative of) the remaining in-vehicle time *t*_*e*_^*ivt*^, plus the required waiting time *t*_*r*+*1,j*_^*wt*^. Hence, the perceived (dis)utility coefficient *β*_*r*+*1,j*_^*ivt*^(*t*) = *β*_*r*+*1,j*_^*wt*^(*t*) = -1.0 and staying utility is evaluated according to (Eq. 10):
  10 $${v}_{i,k,j}\left(r+1\right)=-1\cdot \left({\sum }_{e\in i}{t}_{e}^{ivt}+{t}_{r+1,j}^{wt}\right)$$
  The utility of boarding the first departure *r* involves no extra waiting time (*t*_*r,j*_^*wt*^ = *0*) and the same amount of remaining in-vehicle time *t*_*e*_^*ivt*^ as in [Eq. (10)]. Given that *β*_*r*+*1,j*_(*t*) = − 1.0 , the WTW utility coefficient *β*_*r,j*_^*WTW*^(*t*) acts then as a travel time multiplier due to higher (over)crowding disutility on-board the first departure *r*, which are—on the basis of acquired RTCI - expected upon its arrival at stop *j.* Finally, the boarding utility is calculated as follows (Eq. 11):
  11 $${v}_{i,k,j}\left(r\right)={\beta }_{r,j}^{WTW}\left({t}_{a,k}\right)\cdot {\sum }_{e\in i}{t}_{e}^{ivt}$$
  The formulations in Eqs. 10 and 11 define how boarding and staying utilities are calculated with access to RTCI concerning next vehicle departures from a given stop *j*. Essentially, depending on the trade-off between extra waiting time *t*_*r*+*1,j*_^*wt*^ vs. RTCI-based crowding disutility factor *β*_*r,j*_^*WTW*^(*t*), the staying utility *v*_*i,k,j*_(*r* + 1) may become higher than boarding utility *v*_*i,k,j*_(*r*), resulting with a greater propensity towards staying. In the event that passenger *k* does not access RTCI or has already chosen to skip the previous vehicle, we assume that no WTW is possible anymore and set *β*_*r,j*_^*WTW*^(*t*) = *− *1.0. Passengers will then choose to board the first incoming departure *r*.
  Finally, the choice probability *p*_*a,k,j*_ of passenger *k* boarding the nearest vehicle run *r* at stop *j* is calculated. Since this involves two choice alternatives—i.e., boarding *u*_*a,k,j*_(*r*) vs. staying *u*_*a,k,j*_(*r* + 1)—the choice model in (Eq. 2) reduces down to the binary logit model (Eq. 12):
  12 $${p}_{{a}_{k,j}}=\frac{\mathit{exp}\left({u}_{{a}_{k,j}}\left(r\right)\right)}{\mathit{exp}\left({u}_{{a}_{k,j}}\left(r\right)\right)+\mathit{exp}\left({u}_{{a}_{k,j}}\left(r+1\right)\right)}$$

**Table 1:** RTCI framework, i.e. assumed mapping procedure between volume-capacity ratio vs. 4-level categorical crowding information

RTCI (real-time crowding information)		Volume-capacity ratio with respect to	
Graphical representation	Descriptive interpretation of on-board travel conditions	Seat capacity *K*_*r,s*_^*c*^*/ƞ*_*r*_	Total capacity *K*_*r,s*_^*c*^*/κ*_*r*_
	Severe overcrowding, possible denial-of-boarding risk	> 1.0	≤ 1.0
	Moderate crowding, no seats, but can stand and move freely inside	> 1.0	≤ 0.8
	No standing crowding, individual (10–20%) seats available	> 0.8 and ≤ 1.0	≤ 0.8
	No crowding, more than 50% of seats available	≤ 0.8	≤ 0.8

**Table 2:** WTW thresholds, as a function of available RTCI on crowding levels of the next vehicle departures, based on own stated choice experiments (Drabicki et al. 2022)

WTW (willingness to wait) thresholds—mean values *t*_*r,j*_^*WTW*^ [mins]		RTCI of run *r* *β*_*r,s*_			
				
RTCI of run *r* + *1* *β*_*r*+*1,s*_		0*	0*	0*	
		**9**			
		**10**	**3**		


Values denote the average acceptable waiting time [mins] for the second incoming departure (rows), once passengers decide to skip deliberately the first departure (column) leaving now from the stop, in exchange for difference between crowding levels *β*_*r,j*_(*t*) and *β*_*r*+*1,j*_(*t*)

*No WTW applicable

The queuing process in *BusMezzo* observes the so-called *first-come-first-serve* (FCFS) principle, i.e. passengers are served in their arrival order at the stop. This implies that passengers who form the residual queue at stop *s* after the departure of bus *r*—either due to denied boarding or voluntary (WTW) decision—have the priority in boarding the next bus *r* + 1 over those who will arrive first at the same stop *s* after the departure *t*_*r,s*_ of bus *r*.

### System-wide indicators of bus-bunching

Finally, to evaluate how RTCI provision and the induced WTW behaviour influences the effects of bus bunching, we investigate service performance using two following metrics.

The first metric relates to the aggregate journey experience in PT network and is denoted as passenger welfare *w.* This expresses the total experienced travel utility *u*_*i,k*_ of all passengers *K*_*o,d*_ weighted by the monetary travel time valuation factor *z* (Eq. 13). Assuming fixed OD demand volumes, an increasing welfare value *w* can be interpreted as greater (perceived) travel time savings and therefore an improved overall journey experience.

13 $$w=-z\cdot {\sum }_{k\in {K}_{o,d}}{u}_{i,k}$$

The second metric relates to the regularity (reliability) of PT service operations, which is measured by means of the coefficient of headway variation *cv*_*e*_^*h*^*,* commonly used in bus bunching analysis [e.g. Cats et al. (2011b)]. Coefficient of headway variation *cv*_*e*_^*h*^ is calculated as the ratio between the standard deviation *σ*(*h*_*r,e*_) of service headways observed for runs $r,r+1,...\in L$ along a specific line segment *e*, relative to their mean value (Eq. 14). An increasing value of the *cv*_*e*_^*h*^ parameter will thus imply a greater magnitude of bunching.

14 $$c{v}_{e}^{h}=\frac{\sigma \left({h}_{r,e}\right)}{{h}_{L,0}}$$

## Application and results

### Experimental set-up

**Fig. 2:**
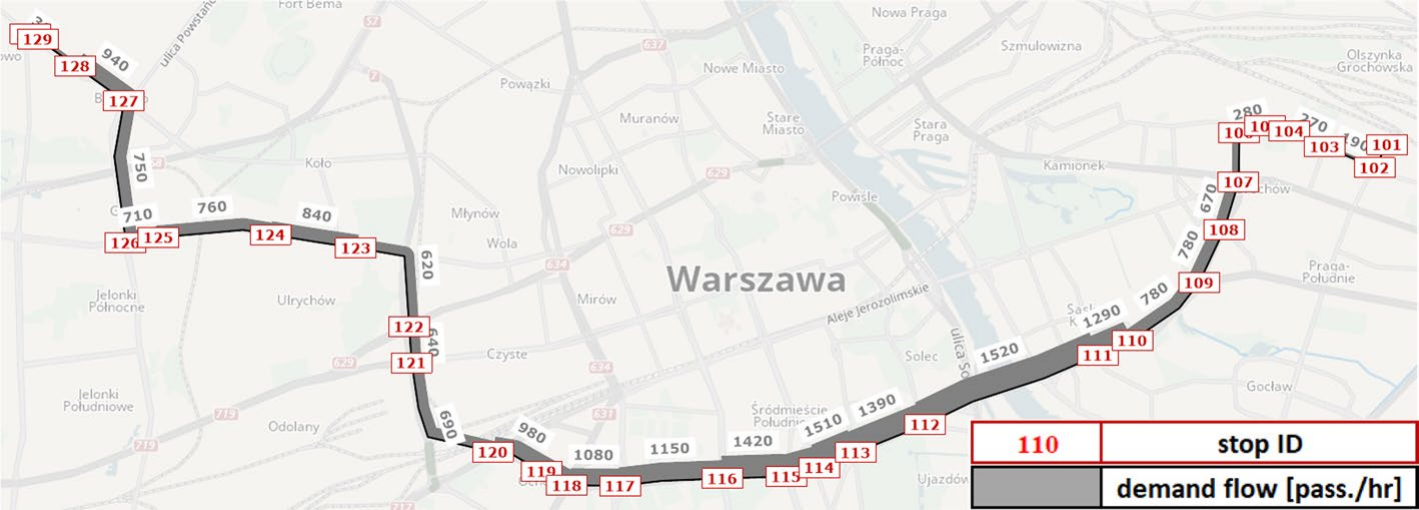
Case-study network: bus line no. 523 in central Warsaw (Poland)

To analyse the potential RTCI effects in relation to bus bunching problem, we introduce the following simulation setup (Fig. 2), (Table 3). It is modelled based on one of the busiest bus lines in Warsaw. Line no. 523 is a cross-city bus service, spanning between western and eastern parts of the city with 30 stops, served by 12 buses per hour. We model the westbound direction, which is particularly busy and susceptible to bunching during the morning peak period. Supply and demand data is based on the Greater Warsaw strategic transport model (MTAW 2017) (Warsaw Municipal Authority 2017). Peak passenger volumes are observable between stops no. 111 and 116, reaching ca. 1500 [pass./h]. The remaining downstream line segments are somewhat less crowded, with cross-sectional passenger volume ranging around 700–1100 [pass./h] up to the terminal stop no. 130.

**Table 3:** Case-study network supply and demand specifications

Input parameter		Value	Input parameter	Value
Dwell time increment *τ*_*r*_				
Boarding		2.0–4.0 [s/pass.]	Total service run time *Σt*_*r,e*_	57 [mins]
Alighting		1.5 [s/pass.]	Total passenger demand *Σq*	3650 [pass./h]
Nominal service headway *h*_*L,0*_		5 [mins]	Vehicle seat capacity *ƞ*_*r*_	60 [pass./veh.]
Initial delay *φ*_*s*_ (*r* + *1,r* + *3,…*)		+ 90 [s]	Vehicle total capacity *κ*_*r*_	150 [pass./veh.]

A key driver of demand–supply variations pertains to the flow-dependent dwell time function as described in previous section by (Eq. 1). To induce bunching in our PT network, we introduce an ‘initial’ service disturbance, whereby alternate bus trips are either dispatched on-time (i.e. dispatching time offset *φ*_*s*_(*r,r* + *2,…*) = 0 [s]) or with an initial delay of *φ*_*s*_(*r* + *1,r* + *3,…*) =  + 90 [s]. Therefore, even though the nominal service headway is equal to *h*_*L,0*_ = 5 [mins], the actual headway at origin stop will oscillate alternately between *h*_*r,e*_ = 3.5 [mins] and *h*_*r*+*1,e*_ = 6.5 [mins]. Consequently, bus service regularity may become substantially disrupted en-route, due to emergence and amplification of bunching effects, which may still persist in downstream network despite decreasing passenger volumes.

Since we run a stochastic PT simulation model, each scenario outputs are reported as an average of 30 simulation replications with a random seed. This number of replications was found to be statistically significant, yielding a maximum allowable error of 3.8%. PT supply is simulated for a period of 3 h (to account for service warm-up and cool-down periods), while PT demand is generated during an intermediate 60-min period, from the 30th to the 90th min of simulation run time.

We analyse and compare scenarios with identical demand and supply and differing in the RTCI availability: the baseline *“no RTCI”* case (i.e. default passenger behaviour without crowding information access); and the *“RTCI”* case (i.e. where passengers utilize crowding information in their boarding decisions). Additionally, in Subsection 3.1.4. below we also introduce intermediate scenarios with variable RTCI penetration rates (i.e. 25%, 50% and 75% of passengers obeying the RTCI). Output passenger welfare estimations are based on monetary time valuations projected according to JASPERS (2015) for the 2021 data. Once weighted across three basic trip categories (business, commuting and leisure trips), these yield an average rate of 34.6 [PLN/h], or ca. 7.5 [EUR/h] (assuming the conversion rate of 4.6 [EUR/PLN]).

The main objective of experiments presented below is to analyse the implications of RTCI provision as a potential mitigation measure against bus bunching, as demonstrated on a real-world bus line model with the proposed RTCI simulation algorithm.

### Emergence of the WTW behaviour

**Fig. 3:**
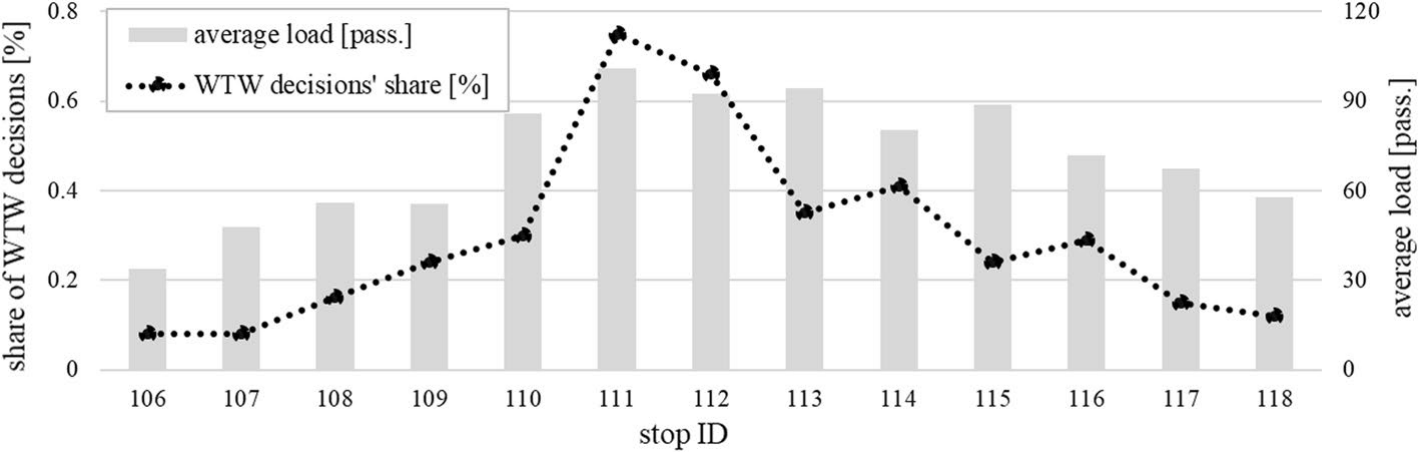
Share of WTW boarding decisions, plotted against the average passenger load, along the central bus corridor segment in the *RTCI* scenario

Differences between scenarios are principally driven by passengers’ boarding decisions. The *no RTCI* scenario employs the default decision making process, where passengers always choose the first arriving bus trip, yet might nevertheless be denied from boarding in the event that capacity limits become binding. In the *RTCI* scenario, crowding information induces variable willingness-to-wait (WTW) probability at PT network stops (Fig. 3). Initially, share of travellers that opt to wait deliberately for a later departure stays low at upstream stops, until stop no. 110, where the RTCI indicates noticeable on-board load variations between approaching bus trips. Consequently, the WTW pattern holds true in 30% of all boarding decisions at that stop. The share of WTW-based decision peaks then at stops no. 111 and 112 around 60–70%. This high probability results from the corresponding RTCI content which shows high overcrowding of the first bus trip (which has already left stop no. 110), and simultaneously, seats available on-board the second bus trip (which is yet at stops no. 108–109). The WTW share plunges then to ca. 40% at stops no. 113–114 and further down to 20–30% thereafter. Despite similar *average* passenger load, the share of WTW decisions from the stop no. 113 onwards becomes successively lower. This is attributable to much more uniform (over)crowding conditions being displayed for *individual*, consecutive bus trips, which in turn reduces the propensity to wait further at the next stops. Once buses exit the high-demand segment, the WTW share reduces to 20% or lower values. In the following subsections, we summarise the main implications of emerging WTW behaviour for demand and supply performance.

### Effects on service performance

**Fig. 4:**
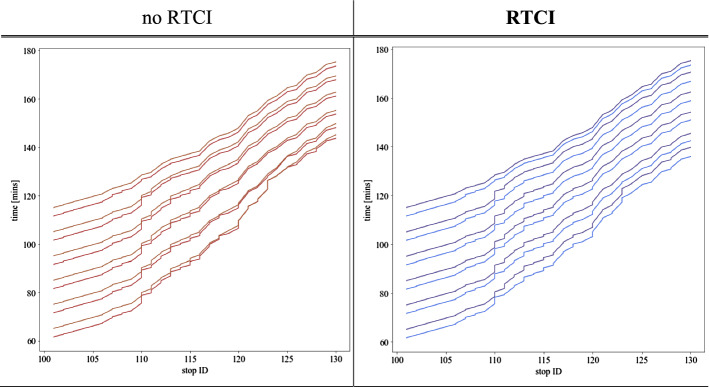
A snapshot of selected bus trips’ space–time trajectories in the *no RTCI* scenario (left) vs. *RTCI* scenario (right)

Figure 4 presents space–time bus trajectories in our case-study network. These reveal an evident bus bunching effect in the *no RTCI* scenario, as the initial dispatching delay eventually propagates into a considerable service disruption. The delayed bus trip is boarded by a larger number of waiting passenger at downstream stops than expected and thereby becomes even more delayed, while the next bus picks up fewer passengers and departs earlier than expected. This effect becomes amplified as high-demand conditions emerge further along the line. Major deteriorations take place in effective service headways *h*_*r,e*_, which become almost double the rate of nominal values (*h*_*L,0*_ = 5 [min]). Eventually, the faster bus ‘catches up’ with the delayed (preceding) bus and ‘full bunching’ effect occurs, observable as bundled bus trajectories in the Fig. 4 (left). From this point onwards, bus service is unable to recover towards regular service pattern and bus trips operate in such staggered combination for the remainder of their trip.

**Fig. 5:**
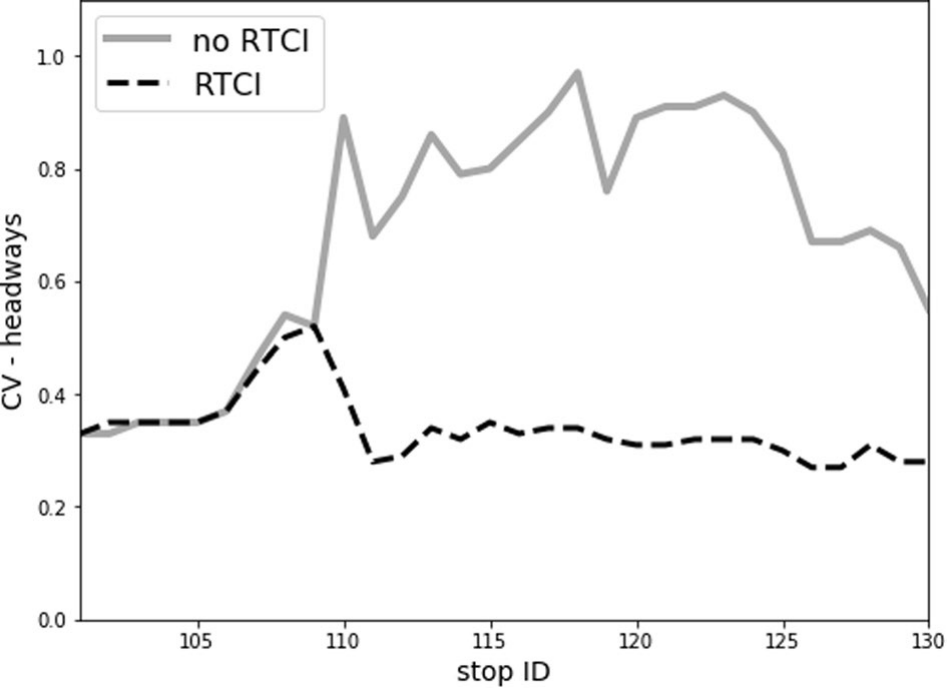
The coefficient of bus headway variation along the line route in the *no RTCI* vs. *RTCI* scenarios, evaluated for peak (intermediate) trips

Conversely, the prevalence of WTW behaviour in the *RTCI* scenario results in a substantially different bus trajectory pattern. As projected in the Fig. 4 (right), this is especially observable from the beginning of high-demand line segment. It is the ‘faster’, less-crowded bus, which undergoes a greater increase in dwell time at stop no. 110, while the ‘slower’ and overcrowded bus departs quicker. Eventually, though full service regularity cannot be restored, a major improvement is reflected in the effective headway *h*_*r,e*_ regularity (Fig. 5). Thus, effective headways between buses are much more favourable in contrast to the *no RTCI* scenario, and no progression towards ‘full’ bunching takes place.

These service regularity patterns are also reflected in the coefficient of variation (CV) of bus headways, plotted along consecutive stops (Fig. 5). In both scenarios, the initial dispatching delay implies a baseline CV rate of ca. 0.3 at the departure from origin stop no. 101. The CV rate increases then gradually, exceeding 0.5 at stop no. 110 where different patterns emerge for both scenarios. Without access to RTCI, high demand conditions cause a substantial increase in CV rate to values of 0.8–1.0 at the next stops—implying a ‘fully bunched’ bus service. Although passenger demand decreases, service regularity is still substantially impeded along the rest of the line, and bus trips arrive at final stop with an average headway CV of ca. 0.6. Conversely, in the *RTCI* scenario, risk of service regularity deterioration is effectively curtailed in the downstream network, notably along the high-demand line segment. The headway CV initially drops down by half from ca. 0.55 at stop no. 109 to 0.27 at stop no. 111 and stabilizes from this point onwards. Henceforth, even despite the rising influence of alighting actions upon dwell times at stops no. 118–130, this does not negatively affect the service regularity. Headway CV hovers around 0.3 and gradually decreases further, down to ca. 0.23 at the final stop. Comparison of both plots (Fig. 5) reveals thus that RTCI availability mitigates the risk of progression towards full bunching and suppresses headway CV at the rates of 0.2–0.3 along the majority of the line, including the most overcrowded conditions.

### Effects on trip loads’ distribution

**Fig. 6:**
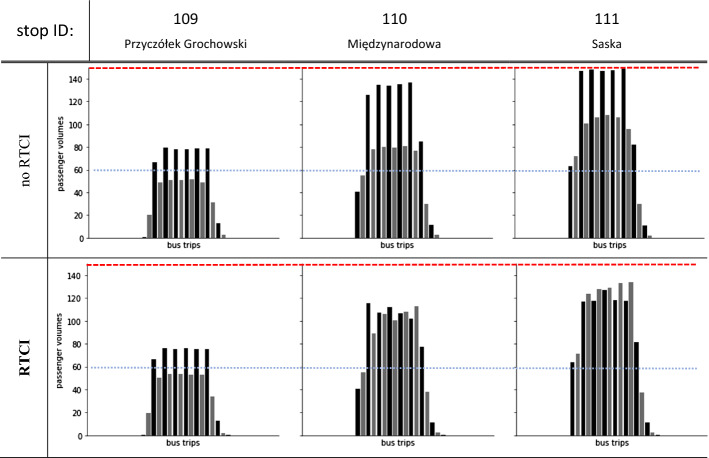
Passenger load distribution on-board consecutive vehicle trips in the *no RTCI* (top) vs. *RTCI* (bottom) scenarios. Selected plots for stops no. 109–111, where the bus line enters high-demand (and thus bunching-prone) segment. Horizontal dashed lines denote bus seat capacity (blue) and crush capacity (red). (Color figure online)

Figure 6 depicts passenger load distribution at selected stops where WTW choice behaviour becomes evident with the emergence of high network demand conditions. Passenger loads of bus trips at stop no. 109 exhibit an analogous pattern (albeit with certain variations), as RTCI does not influence much boarding choices at the initial stops. Then, in the *no RTCI* scenario, it is evident how the feedback between growing bus bunching effect and the default ‘take-the-first-available-departure’ behaviour fosters discrepancies in trip loads’ variations. The alternate bus trips departing from stop no. 110 are either filled up to 90–100% of their capacity limit or have plenty of on-board space available (volume-capacity ratio of 50–60%). At the following stop no. 111, results show a relative increase in on-board loads of the ‘less-crowded’ buses (ca. 70%), which is—however—a consequence of denied-boardings from the fully loaded buses. Such load constellation is yet prevalent along the remaining stops, and even with lower demand in downstream network, variations in bus trip loads are still likely to persist until the final stops.

An opposite pattern is demonstrated in the *RTCI* scenario. Flexibility in boarding choice behaviour results in more equalized load distribution pattern from the stop no. 110 onwards, since the RTCI raises passengers’ awareness of less-crowded buses approaching the stop. In contrast to the *no RTCI* scenario, on-board volumes oscillate in a uniform manner around 70% at every consecutive bus departure. Likewise, with the influx of additional passenger volumes at the stop no. 111, uniformity in vehicle loads’ distribution is preserved, and not a single bus exceeds ca. 90% of its capacity limit. The RTCI-induced WTW choice probability encourages equalization in trip loads at further downstream stops. Its effectiveness becomes somewhat lower, though, with rising impact of alighting actions. Certain load variations emerge along the final stops (between nos. 120 and 130), yet in a much more limited extent compared to the *no RTCI* scenario. Consequently, without RTCI access, load factors of bus trips bounce alternately between full and moderate utilization, whereas with RTCI availability they stabilise within a constrained range.

### Effects on journey experience

Table 4 presents output passenger journey experience results, measured by total travel disutility—i.e., perceived journey time (PJT), as well as by specific PJT components. Globally, RTCI access leads to overall PJT reduction of ca. 5%. Firstly, these changes are mostly attributable to a 6% decrease in the in-vehicle time (IVT) disutility, reflecting more positive on-board comfort experience. Secondly, despite the WTW behaviour encouraging further waiting at the stop, overall waiting time (WT) disutility does not deteriorate in relation to the *no RTCI* scenario. This is attributable to the counteracting effect of reduced waiting times because of improved service regularity. The decrease in total PJT is also intertwined with a 2% lower mean absolute journey time, reflecting that network-wide WTW choices may contribute positively to real-time service performance as well. On average, the PJT changes induced by RTCI are equivalent to welfare benefits of ca. 0.2 EUR (0.9 PLN) per each passenger trip. Once projected in annual terms, total welfare benefits—attainable for this single bus line during a single PM peak hour only—would amount to ca. 223 k EUR (1.0 m PLN).

**Table 4:** Passenger journey experience—comparison between *no RTCI* vs. *RTCI* scenarios. Summary provided both for total perceived journey time (PJT) as well as individual PJT components, including denial-of-boarding disutility

Weighted travel time [h]		No RTCI	RTCI	Rel. change [%]
Waiting time (WT)				
Total		575.9	569.2	− 1.2%
Due to denied boarding		**31.4**	**17.1**	− **45.5%**
Excess (EWT)		271.3	264.7	− 2.4%
In-vehicle time (IVT)—total		1376.0	1290.8	− 6.2%
Perceived journey time (PJT)—total		**1951.9**	**1860.0**	− **4.7%**
Absolute journey time—mean [mins]		19.1	18.7	− 1.8%

In particular, a major benefit of RTCI-induced effects in load distribution and service regularity relates to the substantial, 45% decrease in WT disutility due to denied boarding. Their favourable influence is also reflected in a global 2% decline in excess waiting time (EWT). Both these effects vary locally, though. Passengers boarding at the high-demand stops are more likely to experience longer EWT (up to extra 2–3 [mins] on average), yet in exchange—they gain the most from denial-of-boarding risk lower by as much as 70–100% at selected stops. Otherwise, passengers boarding before the high-demand segment (stops no. 107 to 109) may experience an extra EWT of 1–2 [mins] on average, while those entering downstream of stop no. 120 stand to gain the most from lower WT disutility—being (in turn) an outcome of average EWT shorter by 2–4 [mins] and a wholly mitigated denied-boarding risk.

**Fig. 7:**
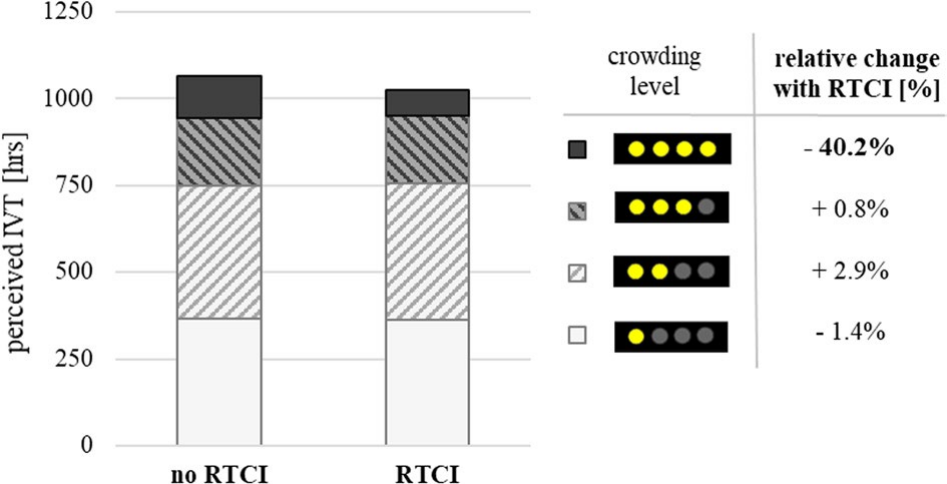
Relative changes in passengers’ on-board comfort experience (i.e. total in-vehicle travel time) in comparison with the *no RTCI* scenario. Distinguished for 4 crowding levels, corresponding to the RTCI categorization scale (Table 1). Total perceived IVT reduces by 7.5% in the *RTCI* scenario

A further inspection of RTCI impact on passengers’ on-board comfort experience is illustrated in Fig. 7. In addition to the aforementioned 6% reduction of total IVT disutility, another major benefit is revealed in total IVT spent by passengers in the highest overcrowding conditions (i.e. RTCI level 4), which is globally lower by 40%. This is accompanied by a modest rise of 2% in journey experience corresponding to seated conditions (RTCI level 1 and 2). In addition to demand-side benefits, such findings imply lower share of massively overcrowded bus trips and thus more efficient capacity utilization.

**Fig. 8:**
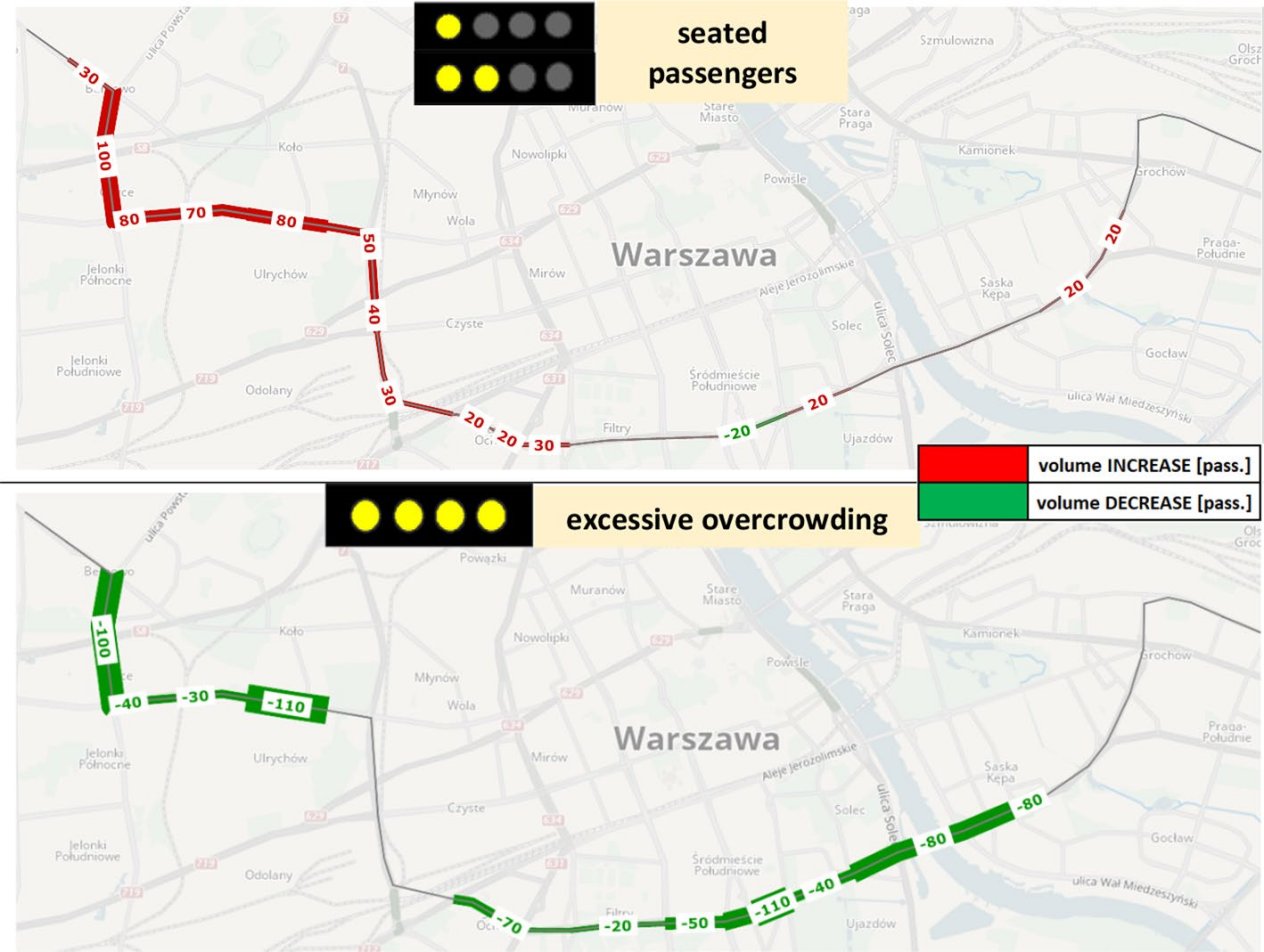
Resultant changes in passenger volumes able to travel seated (top) and avoid excessive on-board overcrowding (bottom) in the *RTCI* scenario

The above findings are also reflected in Fig. 8, depicting changes in passenger volumes subject to different crowding conditions along the line. The upper chart shows a higher number of passengers able to get a seated place, especially right after the high-demand, central line segment (from stops no. 117 and 118 onwards). Volume changes in the *RTCI* scenario range between extra 20 to 100 seated [pass.], i.e. up to 13% of total demand flows. The lower chart indicates a significant drop in passengers experiencing excessive on-board overcrowding in the *RTCI* scenario, which is evident in 2 parts of the network. Firstly, a decrease of 80–110 [pass.] is observable along the highest-demand line segment, as passenger loads are spread evenly across the buses thanks to WTW decisions at consecutive stops. Secondly, improved service regularity along downstream line segments essentially mitigates the on-board overcrowding risk, with up to 110 [pass.] able to travel in less-crowded conditions.

**Fig. 9:**
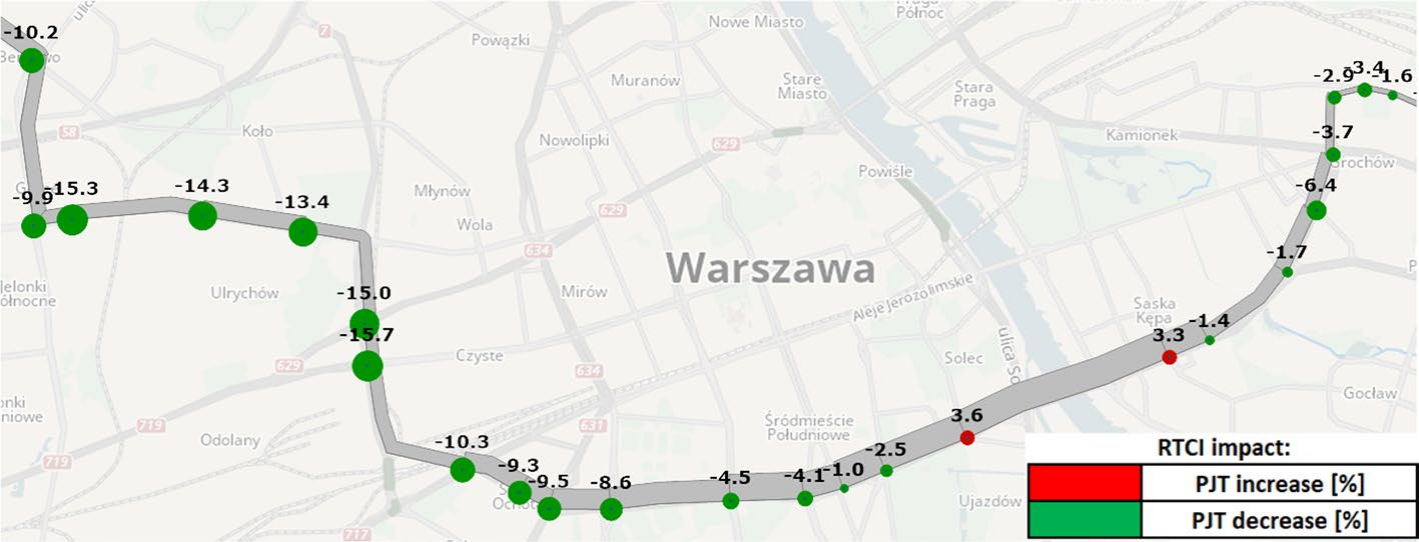
Relative changes (in [%]) in total travel disutility (i.e. total PJT) in the *RTCI* scenario, distinguished for passengers boarding at individual bus stops

To examine the spatial RTCI impact upon travel experience, in Fig. 9 we plot resultant changes in total travel disutility (i.e. PJT), distinguishing them for passengers boarding at various stops. The plot indicates that a vast majority of bus line users stand to gain from ubiquitous RTCI provision and utilization. Those boarding at initial, low-demand stops experience a 3–7% reduction in average PJT, compared to the *no RTCI* scenario. Here, the improved on-board (IVT) utility (experienced later en-route) outweighs the contractions in waiting time (WT) utility (endured at these stops). The only passengers who are likely to experience worse PJT in the *RTCI* scenario—albeit by a mere 3.5%—are those boarding at stops nos. 112 and 113. These stops are located along the peak-demand line segment and rising excess wait time becomes highly relevant for output travel experience at these locations. However, from this point onwards, average PJT savings exhibit a downward trend in the *RTCI* scenario—from 4% for users boarding at stops no. 115 to even 14–16% for those at stops no. 121–125. Passengers boarding downstream enjoy thus relatively the greatest improvements in journey experience and reductions in both IVT and WT disutility. This is, in turn, a favourable outcome of WTW decisions at upstream stops, as they ultimately prevent evolution of major bunching irregularities along the bus line, which are otherwise very prominent in the *no RTCI* scenario.

### Impact of variable responsiveness to RTCI

The above presented *no RTCI* and ‘full’ *RTCI* scenarios assume that real-time crowding information is utilized in boarding decisions by 0% and 100% of passengers, respectively. More realistically, the RTCI utilization will vary depending on the information accessibility, propensity to observe and use the information etc. Therefore, to examine the RTCI effectiveness with variable penetration rate, we extend the above simulation set with *intermediate* scenarios. These are three additional scenarios, where 25%, 50% and 75% of (randomly selected) passengers obey the WTW choice algorithm in their boarding decisions, whereas the remainder follow the default *‘take-the-first-available-bus’* boarding principle. Likewise, outputs for each scenario are extracted from 30 simulation runs with randomized seed.

Table 5 reports changes in experienced travel utility for different RTCI penetration rates, evaluated against the *no RTCI* scenario. It turns out that improvements are already noticeable for the 25% RTCI penetration rate, mainly in terms of waiting time (dis)utility and overall PJT savings equal to 2%. These increase to 4–5% for the RTCI penetration rates of 50% and 75%, when passengers stand to gain the most from reductions in denied-boarding disutility (ca. − 65%), on-board overcrowding (− 50%) excess wait time (− 9%). The overall PJT savings are preserved on the similar level as responsiveness to RTCI approaches the*’full*’ 100% rate. These result then mostly from enhanced on-board (IVT) utility, as ubiquitous response to RTCI leads to limited improvements in waiting time disutility. Nevertheless, these results reveal positive influence of RTCI availability upon travel experience already at low RTCI utilization rates.

**Table 5:** Impact of RTCI penetration rate on passenger journey experiences—changes relative to the *no RTCI* scenario

Travel utility changes vs. *no RTCI* scenario		RTCI penetration rate			
		25%	50%	75%	100%
Perceived journey time (PJT)—total		− **1.9%**	− **4.6%**	− **5.3%**	− ** 4.7%**
Waiting time (WT)					
Total		− 2.2%	− 4.3%	− 3.4%	− 1.2%
Due to denied boarding		− **43.6%**	− **71.2%**	− **64.5%**	− **45.5%**
Excess (EWT)		− 4.6%	− 9.0%	− 7.3%	− 2.4%
In-vehicle time (IVT)					
Total		− 0.9%	− 2.5%	− 4.4%	− 6.2%
Overcrowding (RTCI lvl 4)		− **14.9%**	− **44.8%**	− **52.5%**	− **40.2%**

**Fig. 10:**
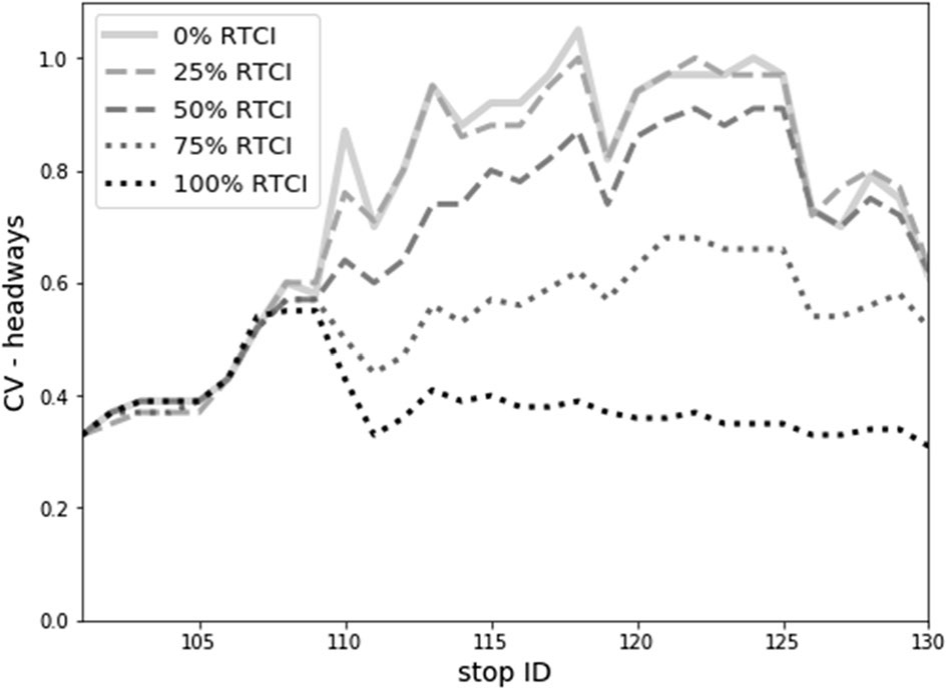
Impact of variable RTCI penetration rate upon coefficient of headway variation, evaluated for peak (intermediate) trips

On the supply side, Fig. 10 depicts the impact of RTCI penetration rate upon service regularity. For the response rates of 25% and 50%, the equalization of bus passenger loads can be already observed, yet notwithstanding—bus bunching persists along the downstream line sections with significant headway variations. Once the RTCI penetration rate is prevalent, however, a much greater and positive impact upon headway regularity is noticeable. For the 75% RTCI penetration rate, maximum headway variation coefficient oscillates at ca. 0.5–0.6. Finally, with universal (100%) response rate, the evolution of bus bunching is effectively suppressed, and headway variations reflect a stable, downward trend. It is thus observable that higher RTCI penetration rates yield an increasingly advantageous impact upon headway regularity in our case-study PT network.

## Discussion

The objective of this study is to investigate the potential application of real-time crowding information (RTCI) for counteracting the bus bunching problem. To this end, we describe the novel RTCI effects upon passengers’ travel behavior using a PT simulation model and analyse their consequences for PT network performance.

The contributions of this study are twofold. The first contribution pertains to the proposed methodology—i.e., an extended passenger path choice algorithm. Its purpose is to simulate how generating and providing RTCI concerning the two nearest vehicle arrivals induces the so-called willingness-to-wait (WTW) for a later bus (vehicle) departure to avoid (reduce) overcrowding among passengers waiting at urban PT stops. Model specification is based on own stated-preference survey findings of the WTW phenomenon among urban PT users (Drabicki et al. 2022). The WTW choice model is implemented within a dynamic agent-based PT assignment model and is thus applicable for analysing the network-wide implications of RTCI provision upon PT demand–supply interactions, travel experience and network performance.

The second contribution relates to its application onto a real-world bus corridor in the Warsaw. Findings reveal how the WTW behaviour incited by RTCI provision might suppress and even reverse the unravelling bunching process. RTCI access has been shown to substantially mitigate service irregularities—as witnessed by headway CV capped at the rates of 0.2 to 0.3—in service conditions which would otherwise spiral into a ‘full-fledged bunching’ state. The evolution of bus bunching was effectively mitigated both along the central, high-demand line segment, as well as in the downstream network dominated by alighting flows (which cannot be influenced by RTCI). Additionally, RTCI utilization resulted in a much more uniform passenger load distribution among consecutive bus trips, especially at high demand stops. This enabled a more efficient capacity utilization with fewer bus trips being alternately overcrowded or underutilized.

Network-wide availability of RTCI on upstream trip loads implied that even as many as 30–70% of passengers opted to wait further at the stop to board a less-crowded bus. The collective dynamics resultant from all the individual, RTCI-induced choices eventually led to a system-wide reduction in total travel disutility (i.e. perceived journey time) of 5%. This stemmed mostly from improved on-board journey comfort across the bus route, and importantly—despite RTCI encouraging the WTW behavior—total waiting disutility did not deteriorate in our case study. This was attributable to the counteracting effect of globally shorter waiting times associated with higher service regularity. Major benefits were especially reflected in lower prevalence of denied boardings and excessive on-board overcrowding—globally by as much as 40%, and locally these risks were eliminated altogether. Such benefits were also attainable for a major share of passengers in the central high-demand network segments, while those travelling in downstream, less-crowded network were additionally more likely to get a seated place on-board (up to extra 13% of total demand flows). Improvements in travel utility were in general experienced by passengers boarding at a vast majority stops in our case-study bus corridor, reaching on average 2% at central line segments, 6% at upstream locations, and even up to 12–16% in the downstream parts of the network. In monetary terms, passenger welfare benefits induced by RTCI provision along this bus corridor would be equivalent to approx. 0.22 m EUR annually (for the PM peak-hour period only). Furthermore, sensitivity analysis has shown that travel experience benefits are already achievable with a limited response rate to RTCI among passengers, with additional improvements in headway regularity once RTCI utilization becomes increasingly prevalent.

To summarise, our findings demonstrate that providing RTCI on upstream trip loads may stimulate a co-operative travel behaviour in form of WTW to reduce (over)crowding, helpful in mitigating the bus bunching problem. Favourably, ubiquitous response in individual boarding decisions across our case-study network was shown to act in a certain *equilibrating* manner, bringing network-wide benefits in terms of improved service regularity and journey experience. To the best of our knowledge, ours is the first study that investigates the impacts of RTCI provision on a real-world bus corridor model, in context of bus bunching and based on empirical (stated-preference) insights into WTW probability among urban PT users. Our results demonstrate the potential efficacy of WTW behaviour with RTCI to become a useful travel demand management strategy in reducing PT disruptions such as bus bunching. Although bunching might not be fully avoidable, simulation results suggest that the behavioural response to RTCI can decelerate and under certain circumstances even halt the negative feedback loop underlying the bunching process. Hence, RTCI offers a *soft* bottom-up bunching mitigation measure which is consistent with and driven by passengers’ aim to maximize travel utility, while simultaneously bringing system-wide performance benefits. Consequently, this can contribute to enhanced PT quality of service and travel experience, either as a sole measure or in conjunction with supply control strategies.

Findings reported in our work shed more light onto the hitherto uninvestigated WTW phenomenon, which might become a common travel behaviour pattern with the future emergence of RTCI solutions in urban PT networks. The development of adequate analytical tools is thus paramount to proper assessment of how advanced passenger information systems (such as the RTCI) can ensure better PT operational efficiency. This is particularly relevant given the increasing importance of passenger overcrowding in urban areas, fostered additionally by the (yet on-going) COVID-19 pandemic crisis (Gkiotsalitis 2021). Hence, RTCI can facilitate and stimulate the better distribution of demand flows across the network (in compliance with specific loading guidelines), possibly also in lower crowding conditions, and help passengers make more informed, crowding-aware travel choices (Adam et al. 2020).

The above mentioned RTCI benefits will be only achievable if information disseminated is deemed reliable and trustworthy for prospective passengers. This underlines the need for ensuring high-quality, accurate crowding information, possibly in conjunction with demand-anticipatory techniques (similarly to car traffic congestion information). The presented model specification can be extended in the future to account for different crowding prediction rules, and to evaluate their accuracy and network consequences.

Future research may investigate—either analytically or by means of simulation—how RTCI affects bus bunching under various conditions, depending on e.g. supply parameters (service headway, system capacity), dwell-time functions (and boarding regimes such as mingling etc.), network and demand configuration etc. Among these, studies may consider situations where several subsequent departures might be of interest, and possibly—include departures from multiple lines as well. The prevalence of advanced passenger information solutions may encourage the tendency to consider multiple travel options simultaneously, particularly in high-frequency urban PT, though travellers’ bounded rationality and information acquisition and processing limitations need to be taken into account. Additionally, comparison and combination with other PT control strategies will be crucial to fully understand whether the WTW with RTCI can be synergic or interchangeable with strategies such as holding and expressing. Future work may also examine how pre-trip RTCI availability may affect the WTW and induce greater departure-time adjustments, as in our study we considered localized (stop-level) RTCI access only. Finally, real-world applications will allow for calibrating and validating the details of RTCI generation and its utilization by passengers.

## Sets

*G* (*S*, *E*): PT network directed connected graph
*S*: Set of nodes (stops) *s*
*E*: Set of links (line segments) *e*
*L*: Line, i.e. a set of runs (trips, departures) *L* = {*r*_*1*_, *r*_*2*_, *… r*_*n*_}, serving an ordered sequence of stops *s*_*1*_, *s*_*2*_*,* …, *s*_*m*_
*A*: Set of actions *a*
*A’*: Subset of complementary actions, i.e. *A’* = *A\*{*a*}
*I*_*a*_: Set of paths *i*, associated with undertaking an action *a*
*K*_*o,d*_: OD flow, i.e. set of passengers *k*_*od*_ travelling from the origin *o* to the destination *d*
*K*_*r,s*_^*a*^: Passenger load alighting from the run (trip) *r* at the stop *s*
*K*_*r,s*_^*b*^: Passenger load boarding the run (trip) *r* at the stop *s*
*K*_*r,s*_^*c*^: Passenger load on-board the run (trip) *r,* recorded at the departure from stop *s*

## Variables and constants

*s*: Node (stop) between consecutive links *e*^*−*^ and *e*^+^
*e*: Link (line segment) between consecutive tail stop *s*^*−*^ and head stop *s*^+^
*r*: Run (trip, departure) belonging to line $r\in L$
*a*: Action available at node *s*
*a*_*k,s*_: Action chosen by passenger *k* at node *s*
*i*: (Downstream) path from node (stop) *s* to the destination *d*: *i* = {*s*, *s* + *1,* …, *d*}
*k*_*o,d*_: Passenger (agent) *k* belonging to the OD flow *K*_*o,d*_
*o*: Origin node
*d*: Destination node
*h*_*L,0*_: Scheduled dispatching headway of consecutive trips of a line *L* from the origin stop *s*_*0*_
*h*_*r,e*_: Actual headway between consecutive trips *r*, *r* + 1 along the line segment *e*
*φ*_*s*_(*r*): Dispatching time offset of run *r* from stop *s*, with respect to nominal headway *h*_*L,0*_
*t*_*r,e*_: Riding time of trip *r* along line segment *e*
*t*_*r,s*_: Dwell time of trip *r* at stop *s*
*t*_*a,k,s*_: Decision time instance of action *a* by passenger *k* at decision point *s*
*p*_*a,k,s*_: Choice probability of action *a* by passenger *k* at decision point *s*
*u*_*a,k,s*_: Total (expected) utility of action *a* considered by passenger *k* at decision point *s*
*u*_*i,k*_: Total (experienced) utility along path *i* for passenger *k*
*u*_*i,k,s*_: Total (expected) utility of path *i* for passenger *k* at decision point *s*
*v*_*i,k,s*_: Systematic part of (expected) utility of path *i* for passenger *k* at decision point *s*
*ε*_*k*_: Random error part of (expected) utility for passenger *k*
*t*_*e*_^*ivt*^: (Expected) in-vehicle travel time of trip segment *e*
*t*_*e*_^*wt*^: (Expected) wait time at the tail stop *s*^*−*^ of trip segment *e*
*t*_*e*_^*wkt*^: (Expected) walk time to the tail stop *s*^*−*^ of trip segment *e*
*n*_*i*_^*tr*^: (Expected) number of transfers along the path *i*
*β*_*e*_^*x*^: (Expected) co-efficient of utility component *x* at the trip segment *e*
*z*: Monetary valuation factor of travel time *t*
*β*_*r*__*,s*_: Recorded RTCI level (i.e. 1-to-4 scale) of run *r* at the departure from stop *s*
*β*_*r*__*,j*_(*t*): Generated RTCI value of trip *r* at the downstream stop *j,* valid at time *t* ($s,j\in L$: *j* > *s*)
*β*_*a*_^*WTW*^: WTW crowding multiplier at the boarding action instance *a*
*δ*_*a*_^*WTW*^: WTW (willingness to wait) decision instance

## Inputs

*ƞ*_*r*_: Seat capacity of vehicle run *r*
*κ*_*r*_: Crush capacity of vehicle run *r*
*q*_*o,s*_: Origin demand inflow rate [pass./h]
*q*_*d,s*_: Destination demand outflow rate [pass./h]
*τ*_*r*_^*b*^: Dwell-time increment rate per passenger boarding the vehicle run *r*
*τ*_*r*_^*a*^: Dwell-time increment rate per passenger alighting from the vehicle run *r*
*t*_*r,s*_^*WTW*^: WTW threshold, i.e. mean acceptable waiting time for the next run *r* + 1 due to arrive at stop *s*

## Output indicators

*w*: Passenger welfare, i.e. generalized passenger travel cost in monetary terms
*cv*_*e*_^*h*^: Coefficient of headway variation, recorded along the line segment *e*
